# High Fat Diet Inhibits Dendritic Cell and T Cell Response to Allergens but Does Not Impair Inhalational Respiratory Tolerance

**DOI:** 10.1371/journal.pone.0160407

**Published:** 2016-08-02

**Authors:** Angela Pizzolla, Ding Yuan Oh, Suzanne Luong, Sara R. Prickett, Darren C. Henstridge, Mark A. Febbraio, Robyn E. O’Hehir, Jennifer M. Rolland, Charles L. Hardy

**Affiliations:** 1 Department of Immunology, Monash University, Melbourne, VIC 3004, Australia; 2 Department of Allergy, Immunology & Respiratory Medicine, Alfred Hospital and Monash University, Melbourne, VIC 3004, Australia; 3 Baker IDI, Melbourne, VIC 3004, Australia; GERMANY

## Abstract

The incidence of obesity has risen to epidemic proportions in recent decades, most commonly attributed to an increasingly sedentary lifestyle, and a ‘western’ diet high in fat and low in fibre. Although non-allergic asthma is a well-established co-morbidity of obesity, the influence of obesity on allergic asthma is still under debate. Allergic asthma is thought to result from impaired tolerance to airborne antigens, so-called respiratory tolerance. We sought to investigate whether a diet high in fats affects the development of respiratory tolerance. Mice fed a high fat diet (HFD) for 8 weeks showed weight gain, metabolic disease, and alteration in gut microbiota, metabolites and glucose metabolism compared to age-matched mice fed normal chow diet (ND). Respiratory tolerance was induced by repeated intranasal (i.n.) administration of ovalbumin (OVA), prior to induction of allergic airway inflammation (AAI) by sensitization with OVA in alum i.p. and subsequent i.n. OVA challenge. Surprisingly, respiratory tolerance was induced equally well in HFD and ND mice, as evidenced by decreased lung eosinophilia and serum OVA-specific IgE production. However, in a pilot study, HFD mice showed a tendency for impaired activation of airway dendritic cells and regulatory T cells compared with ND mice after induction of respiratory tolerance. Moreover, the capacity of lymph node cells to produce IL-5 and IL-13 after AAI was drastically diminished in HFD mice compared to ND mice. These results indicate that HFD does not affect the inflammatory or B cell response to an allergen, but inhibits priming of Th2 cells and possibly dendritic cell and regulatory T cell activation.

## Introduction

The incidence of obesity has risen in recent decades to reach epidemic proportions worldwide [1]. Co-morbidities associated with obesity include metabolic diseases, particularly type II diabetes mellitus, cardiovascular disease, cancer and chronic inflammatory diseases like non-allergic asthma [2]. Asthma is a chronic inflammation of the airways characterized by reversible airway constriction and bronchospasm, which can be broadly divided into allergic or non-allergic, depending on the nature of the asthmatic trigger. Atopic sensitization, an inherited predisposition to synthesize specific IgE to common environmental aeroallergens, is a major risk factor for allergic asthma. Obesity is a recognized risk factor for non-allergic asthma [3,4], but the link with allergic asthma is less clear, with several studies failing to observe a correlation between obesity and allergic asthma/atopy [5,6]. However, some studies have identified obesity as a risk factor for atopy [7], raising the possibility that being overweight plays a role in the establishment of an allergic response.

It has been hypothesized that allergic asthma results from an impaired ability to develop respiratory inhalational tolerance to allergens [8]. This mechanism has been extensively studied in animal models of asthma, where it is possible to induce respiratory inhalational tolerance by exposing animals to allergen in the absence of adjuvant prior to sensitization and challenge with the same antigen [9]. It has been suggested that obesity increases the risk of asthma and atopy because it induces a status of chronic low-grade inflammation, with decreased immunological tolerance towards antigens [10]. We sought to determine whether a high fat diet (HFD) affects the induction of respiratory inhalational tolerance and the immunological response to allergens.

In a pilot experiment, we observed that feeding mice a HFD for 8 weeks showed a tendency for impaired activation of dendritic cells (DC) and regulatory T cells (Treg) following exposure to allergen in the absence of adjuvant, during the induction of respiratory tolerance. HFD also reduced allergen-specific Th2 cytokine production and DC activation following sensitization and challenge with allergen, which induced allergic airway inflammation (AAI). Despite these effects on pulmonary immune function, respiratory tolerance did not decrease lung inflammation and IgE production in mice fed with HFD. Collectively, our data suggest that a HFD has a tendency to impair the response of airway immune cells to allergens, but does not alter the capacity to develop respiratory tolerance.

## Methods

### Mice

Female C57BL/6 were sourced from Walter and Eliza Hall Institute (WEHI) or Monash Animal Research Platform (Melbourne, Australia) and imported into the Precinct Animal Centre or Monash Intensive Care Unit at the Alfred Medical Research and Education Precinct (AMREP, Melbourne). AMREP animal ethics committee approved all experimental procedures (E/0924/2010M).

### Diets

Normal chow diet (ND) was either irradiated mouse cubes (Barastoc WEHI, Ridley AgriProducts, Melbourne, Australia) or irradiated rat and mouse diet (Specialty Feeds, Glen Forrest, Australia) with both diet having similar nutritional parameters. HFD was a semi-pure diet composed of casein, sucrose, wheat starch, dextrinized starch, cellulose, canola oil, lard, vitamins and minerals (Specialty Feeds). Both diets were composed of mixed cereals, canola oil, meat or fish meal, vitamins and minerals and had similar nutritional parameters (S1 Table).

### Echo magnetic resonance imaging

Mouse lean and fat mass were measured by quantitative nuclear magnetic resonance as previously described [11] (qNMR; Echo Medical System, Houston, TX).

### Microbiota analysis

DNA from fecal samples was extracted using the ISOLATE fecal DNA kit (Bioline, Sydney, Australia). 16S regions were amplified using the Ion 16S Metagenomics kit and sequenced using the Ion PGM Sequencing 400 kit on the Ion PGM platform at the Monash Health Translation Precinct Medical Genomics Facility. Data were analyzed using the Ion Reporter software (all from Life Technologies, Carlsband, CA) and GraphPad Prism.

### Cellular bioenergetics measurements

Lung cells were prepared as described below. Oxygen consumption rate (OCR) and extracellular acidification rate (ECAR) were measured using the Seahorse XF^e^96 Extracellular Flux Analyser and the XF cell mitochondrial stress test kit assay according to manufacturer’s instructions (Seahorse Biosciences, Billerica, MA). 3 x 10^5^ lung cells, prepared as described below, were resuspended in modified DMEM (XF modified DMEM, Seahorse Biosciences) (pH 7.4) supplemented with 1 mM pyruvate and 11 mM glucose for the mitochondrial stress test (mitochondrial function). Following basal energetic or respiration measurements, cells were sequentially treated with 1 μM oligomycin (ATP synthase inhibitor), 0.5 μM carbonylcyanide ptrifluoromethoxyphenylhydrazone (FCCP, proton ionophore), 1 μM antimycin A (complex III inhibitor) and 1 μM rotenone (complex I inhibitor). Mitochondrial function parameters of basal respiration (difference in OCR before and after antimycin A/rotenone treatment), oxidative ATP turnover (difference between OCR following oligomycin and before any treatment), maximal respiratory capacity (difference between OCR following treatment with FCCP and antimycin A/rotenone) and spare respiratory capacity (difference between OCR following treatment with FCCP and prior to any treatment) were determined.

### Glucose tolerance test

6 h-fasted mice were given 1g/Kg dextrose and blood glucose concentrations measured as described previously [12].

### NMR analysis

To measure the concentration of metabolites in the fecal samples, the following protocol was used. Approximately 70 mg of solid material was transferred into an MP-Bio 1.5 mL O-ring tube containing 1.4 mm diameter spherical ceramic lysing beads. 1 mL D_2_O (containing 1% NaCl) was added to the sample which was homogenized in a Precellys-Cryolis homogenization device for 3 x 45 seconds at ambient temperature. Samples were then centrifuged at 14 x 10^3^ rpm at ambient temperature for 5 min to pellet solids. After thoroughly washing and removing excess Milli-Q water from Millipore Amicon-4 4mL filter unit (3kDa cut-off), cellulose membrane), samples (extracted from solids or neat liquids) were centrifuged at 3000g at 4°C until more than 250 μL had passed through. 250 μL of filtered sample was collected and 250 μL D_2_O was added. Samples were mixed and then 300 μL of chilled deuterated chloroform (CDCl_3_) and 200 μL of chilled deuterated methanol (CD_3_OD) were added. Samples were vortexed vigorously and incubated at 4°C for at least 10 minutes. Samples were then centrifuged at 14 x 10^3^ rpm at 4°C for 10 min and a 440 μL aliquot of upper polar/aqueous phase was collected. To this was added 200 μL of 200 mM Na_3_PO_4_·D_2_O and 60 uL of 5 mM DSS (2,2-dimethyl-2-silapentane-5-sulfonic acid) in D_2_O. Samples were carefully mixed and transferred to 5 mm NMR tubes. NMR was run at 25°C on a Bruker-Biospin Avance 800 MHz spectrometer equipped with a 5-mm triple resonance cryoprobe. All samples were locked to D_2_O and gradient shimmed. Data were collected over 64,000 data points and 512 scans. A one-dimensional nuclear Overhauser effect spectroscopy (NOESY) pulse sequence was used with a T1 of 1.5 s and a mixing time of 50 ms, and the transmitter frequency offset was optimized to coincide with the HDO signal that was presaturated during both the recycle time and mixing time. Spectra were processed and metabolites quantified with Chenomx NMR Suite software package.

### Allergen instillation and immunization

Ovalbumin (OVA, Grade 5, Sigma-Aldrich, St Louis, MO) was dissolved in PBS and sterile filtered. The endotoxin level of the OVA was 40EU/100μg as determined by the LAL QCL-1000 Assay for Endotoxin Determination (Lonza, Walkersville, MD). Respiratory tolerance was induced by instilling 100 μg OVA in 50 μl saline (sodium chloride intravenous infusion BP, 0.9%, Pfizer Australia Pty Ltd) i.n. for 5 consecutive days (day 1–5). Mice were sensitized i.p. with saline or OVA (50 μg) in a solution of 10% aluminum hydroxide (Sigma-Aldrich) in saline on day 15 and challenged via i.n. instillation of saline or OVA (25 μg/50 μl saline) on days 25–28.

For tracking OVA uptake, mice were anaesthetized with Ketamine and Xylazine i.p. and given 50 μg of OVA Alexa Fluor 594 conjugate (OVA-AF594, Molecular probes, Life Technologies) in 100 μl sterile PBS intratracheally (i.t.). The endotoxin level of OVA Alexa Fluor 594 waS125 EU/100 μg (determined as above).

### Tissue sampling and cell isolation

BAL fluid was collected from lungs 24 h after the last OVA challenge. Collection and preparation of blood and BAL were as described [13]. Lung and MLN were collected aseptically and finely chopped with a blade or tissue chopper (McIlwain Tissue Chopper, Mickle Laboratory Engineering Ltd, Surrey, UK). The tissues were digested with collagenase IV (Worthington Biochemical, Lakewood, NJ) and DNAse I (Sigma-Aldrich) as described previously [14]. To determine cytokine production, LN cells were stimulated with OVA (25 μg/ml) or medium and cultured for 96 h in 96-well U well plates (Cellstar, Greiner Bio-One, Frickenhausen, Germany).

### Cytokines detection

Levels of IL-5 and IL-13 in MLN cell culture supernatants were measured by specific ELISA (eBioscience, San Diego, CA) as per manufacturer’s instructions. Concentrations of cytokines in BAL fluid were measured with the Bio-plex Pro mouse cytokine 23-plex assay (Biorad, Hercules, CA), with custom made addition of IL-33, and performed according to manufacturer’s instruction. Analysis of the results was performed in Graph Pad Prism. TSLP concentration in BAL fluid and IL-17 concentration in serum were determined with mouse TSLP ELISA read-set-go and mouse IL-17A ELISA read-set-go respectively, both from eBioscience (San Diego, CA), according to manufacturer instructions. Standard curves and analysis of the results were performed with Graph Pad Prism.

### Airway hyperreactivity (AHR)

AHR was performed as described previously [15].

### Flow cytometry

To block Fc receptors, cells were incubated with purified anti-mouse CD16/CD32 (BD Pharmingen, Franklin Lakes, NJ) for 10 min at RT prior to Ab staining. Ab staining was performed for 20 min on ice. Cells were fixed in 2% paraformaldehyde in PBS and data acquired on a LSRFortessa using FACSDiva v6.2 software (BD Biosciences, Franklin Lakes, NJ). Data were analyzed using FlowJo v10 (TreeStar Inc., Ashland, OR). Positive staining was determined by comparison with the isotype control or Fluorescence Minus One control.

The following anti-mouse Abs were used to stain for DC: PE anti-PDL2 (CD273, Ty25), AF700 anti-CD11b (M1/70), Brilliant Violet 605 anti-CD86 (GL-1), biotin anti-CD103 (2E7), Brilliant violet 650 streptavidin, FITC anti-CCR7 (4B12), PE anti-mouse PDL1 (10F.9G2), BV605 isotype control rat IgG2a and PE isotype control rat IgG2a 400508 were all from Biolegend. PE anti-ICOSL (HK5.3), PerCP-eF710 anti-CD317 (PDCA-1, eBio927), Pe-cy7 anti-F4/80 (BM8), APC-anti-CD11c (N418), APC-eFluor 780 anti-MHCII (M5/114.15.2), Pacific Blue anti-CD45R (RA3-6B2) were all from eBioscience. Live/dead fixable Aqua dead cell stain kit was from Molecular Probes. PE-CF594 anti-Siglec-F was from BD Pharmingen.

For staining of Treg the following Abs were used: V450 anti-mouse CD4 (BD Horizon, RM4-5, BD Biosciences), APC-eFluor780 anti-CD25 (PC61.5, eBioscience), AF700 or PE anti-Nrp-1 (761705, R&D systems, Minneapolis, MN, USA), PE/cy7 anti-GITR (CD357, DTA-1, eBioscience), PercP-eFluor710 anti-ICOS (15F9, eBioscience), Live/dead fixable Aqua dead cell stain kit (Molecular Probes). After fixation and permeabilization with FoxP3 staining buffer set (eBioscience), cells were stained with APC anti-Foxp3 (FJK-16s, eBioscience) and PE anti-IL10 (JES5-16E3, eBioscience).

### DC and macrophages OVA presentation assay

DC were isolated from digested MLN enriched with the EasyStep Mouse Pan DC enrichment kit (19728, Stemcell Technologies) following the manufacturer’s instructions but using ¼ of the recommended reagents. DC were then purified to 99% purity by sorting on BD Influx or FACSAria (BD Bioscience) using purified anti-mouse CD16/CD32 (2.4G2, BD Pharmingen), APC-anti-CD11c (N418, eBioscience), APC-eFluor 780 anti-MHCII (M5/114.15.2,eBioscience) and Live/dead fixable Aqua dead cell stain kit (Molecular Probes). DC were gated from singlet and live cells. Lung cells were digested as above and enriched with NycoPrep 1.077 (Axis-Shield). Enriched lymphocytes and monocytes were then stained with the same Abs as for MLN plus PE-CF594 anti-Siglec-F (E50-2440, BD Pharmingen) and sorted with BD Influx or FACSAria (BD Bioscience). DC were defined as in S1 Fig, macrophages were defined as in S1 Fig adding a gate on SiglecF^+^ cells. 10,000 DC and macrophages were pulsed with OVA in complete medium for 2 h at 37°C at 5% CO_2_ in a V-bottom sterile plate (Corning) and then washed three times with complete medium before adding 50,000 CD4^+^CD25^-^ OT-II T cells. OT-II CD4^+^ CD25^-^ T cells were isolated from LN and spleen of OTII female mice, by negatively enriching by magnetic bead selection: cells were stained with hybridoma supernatant containing rat anti-mouse mAbs against CD11b (M1/70), F4/80, Gr1 (RB6-8C5), MHCII (M5/114), CD8 (53-6-7), erythrocytes (Terr/119), all sourced from WEHI (Melbourne, Australia), and magnetic goat anti-rat IgG beads (Biomag). Non-stained cells were separated on a DynaMag-5 magnet (Life Technologies). Cells were stained with purified anti-mouse CD16/CD32 (2.4G2, BD Pharmingen), V450 anti-mouse CD4 (BD Horizon, RM4-5, BD Biosciences), PE anti-mouse CD25 (7D4, BD Pharmingen) and Live/dead fixable Aqua dead cell stain kit (Molecular Probes), the cells were sorted for CD4^+^CD25^-^ on BD Influx or FACSAria (BD Bioscience) up to 99% purity. CD4^+^CD25^-^ T cells were stained with CFSE prior to being added to the APC.

After 84 h culture, cells were stained with purified anti-mouse CD16/CD32 (2.4G2, BD Pharmingen), V450 anti-mouse CD4 (BD Horizon, RM4-5, BD Biosciences), Live/dead fixable Aqua dead cell stain kit (Molecular Probes), PE anti-mouse TCR Vα2 (B20.1, Biolegend), PE-Cy7 anti-mouse PD1 (RMPI 1–30, Biolenged) and APC anti-Foxp3 (FJK-16s, eBioscience) before acquisition on BD LSRFortessa (BD Biosciences).

### Statistics

Data were analyzed using Mann-Whitney’s test when comparing two groups or Kruskal-Wallis followed by Dunn’s post-test when comparing more than two groups. When comparing data from different times or stimulations, 2-way ANOVA was used. For the gut microbiota and metabolite analyses, unpaired t-test with Welch’s correction for non-homogeneity of variance and Bonferroni’s correction for multiple hypothesis testing was used. GraphPad Prism v6 (La Jolla, CA) was used for all analyses.

## Results

### HFD alters gut microbiota and metabolites, and alters glucose metabolism of lung cells

**Fig 1:**
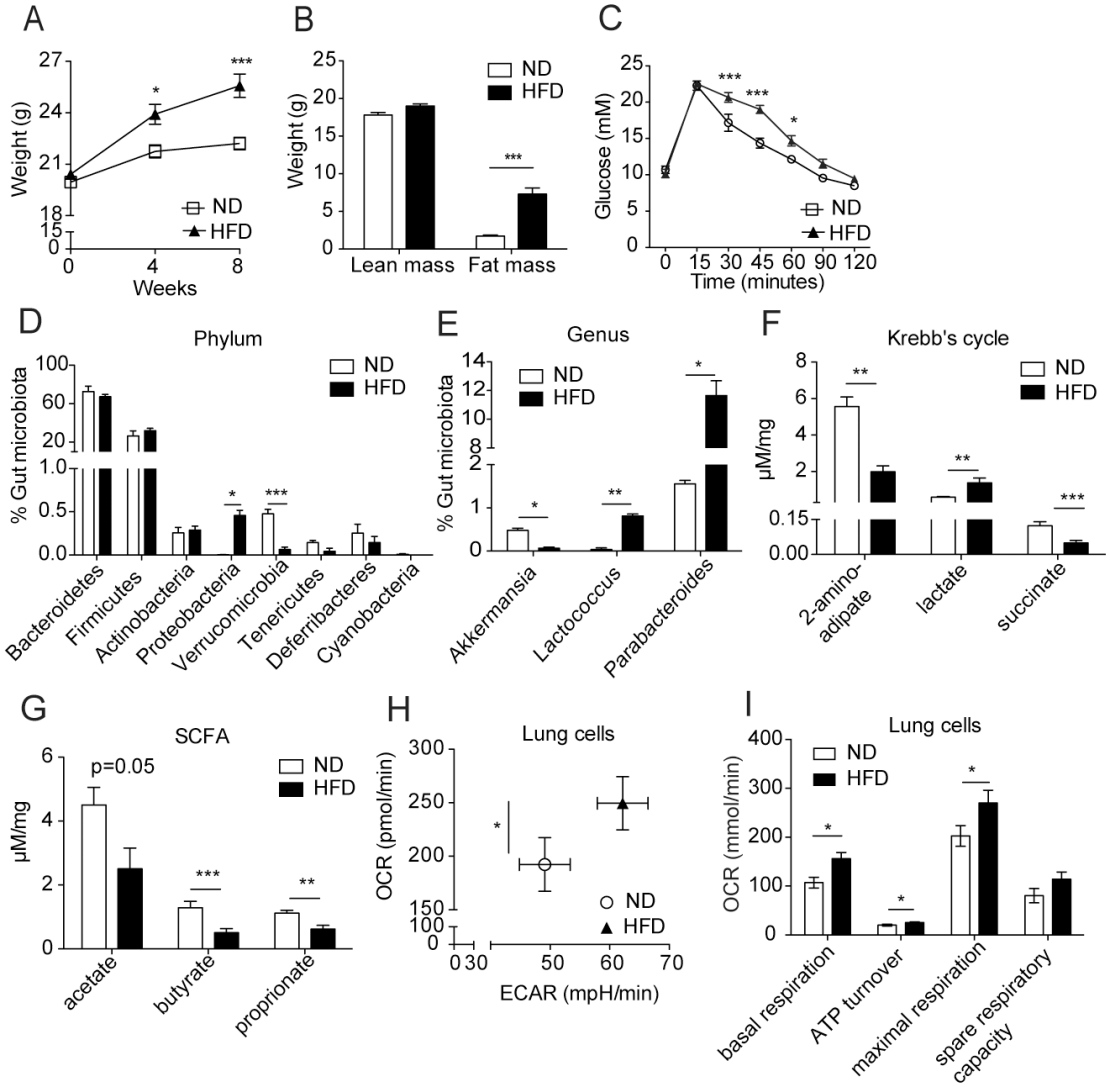
High fat diet caused weight gain and metabolic changes in mice. (A) Weight of mice fed on normal chow diet (ND) or high fat diet (HFD) for 8 weeks. N = 24 mice/group, pooled data from 2 independent experiments. All following data are determined after 8 weeks of feeding. (B) Lean and fat mass. N = 12 mice/group. (C) Blood glucose concentration during glucose tolerance test. N = 12 mice/group. Data in A, B and C were analyzed using 2-way ANOVA. (D-E) Characterization of gut microbiota in ND and HFD mice: phyla (D) and the relevant genus (E), N = 5/group, analyzed using unpaired t-test with Welch’s and Bonferroni’s corrections. (F-G) Concentration of Kreb’s cycle metabolites (F) and short chain fatty acids (SCFA; G) in fecal samples of ND and HFD mice. N = 7-17/group. Pool of 2 independent experiments is shown. Data were log-transformed and analyzed using unpaired t-test with Welch’s and Bonferroni’s corrections. (H) Extracellular acidification rate (ECAR) and oxygen consumption rate (OCR) at basal condition of lung cells. (I) Lung cells OCR of basal respiration, ATP turnover, maximal respiration and spare respiratory capacity. N = 5 mice/group, analyzed using unpaired t-test. *P<0.05, **P<0.01, ***P<0.001. Mean ± SEM are indicated. The experiment was performed once unless otherwise noted.

Six-8 week old C57BL/6 mice were fed HFD or ND for 8 weeks [16,17] and as expected, total body (Fig 1A) and fat (Fig 1B) mass of mice fed with HFD was increased compared with mice fed ND. HFD-fed mice became glucose intolerant (Fig 1C), a symptom of metabolic syndrome. Next, we analyzed the gut microbiota and metabolites by sequencing the 16S ribosomal DNA and measuring the levels of volatile compounds from fecal samples. HFD markedly decreased the percentage of the phylum *Verrucomicrobia* and, within this phylum, the genus *Akkermansia* (Fig 1D and 1E), known to inversely correlate with body weight and when absent, partially mediates HFD-induced metabolic disorders [18]. The increase in *Lactococcus* (*Firmicutes* phylum), exclusive producers of lactate, and *Parabacteroides* (*Bacteroidetes* phylum), fermenting bacteria, in HFD-fed mice was consistent with the increase in lactate detected in the fecal samples of HFD mice compared to ND mice (Fig 1E and 1F). Measuring metabolites in the gut, we found that HFD decreased the fecal concentration of several fermentation products, except for lactic acid (Fig 1F and 1G).

HFD induces metabolic changes at the cellular level [19] and recently the metabolic activity of immune cells has been related to their function [20]. However, to our knowledge, the effect of HFD on the metabolism of lung cells has not been investigated. The metabolic activity of lung single cell suspensions was measured as their capacity to metabolize glucose: this was assessed *in vitro* by measuring the oxygen consumption rate (OCR), and the extracellular acidification rate (ECAR). Lung cells from HFD mice showed higher oxygen consumption compared to cells from ND mice (Fig 1H), as well as higher basal and maximal respiration and ATP turnover (Fig 1I), therefore increasing the capacity to metabolize glucose. Overall, HFD consumption for 8 weeks induced significant weight gain, altered microbiota composition and concentration of metabolites in the gut, and increased the metabolic capacity of lung cells.

### Analysis of DC and Treg activation after intranasal ovalbumin administration

**Fig 2:**
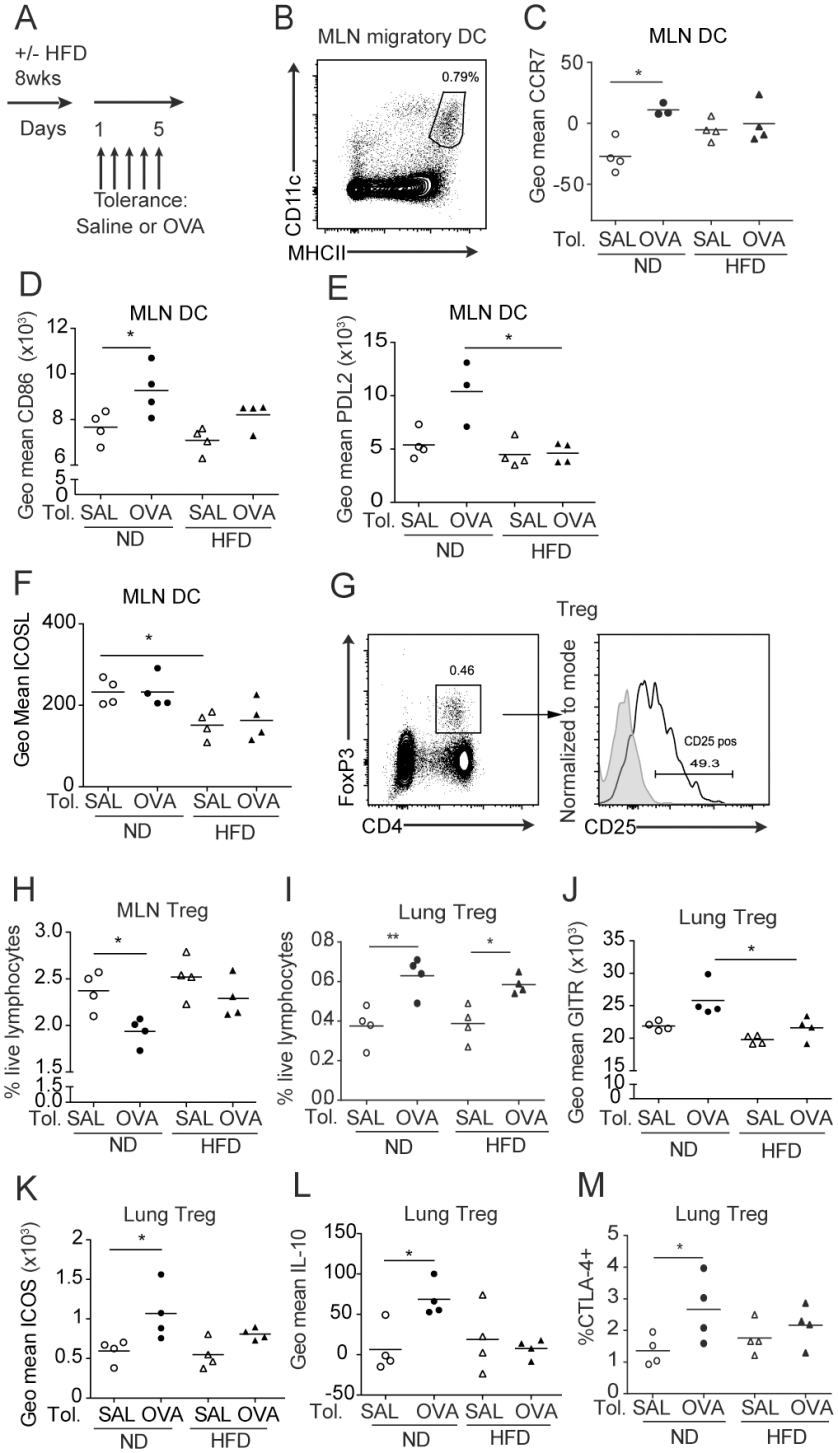
Analysis of activation of MLN DC and lung Treg after intranasal OVA. (A) Experimental design for diet regimen and induction of respiratory tolerance via intranasal administration of saline or ovalbumin (OVA). (B) Gating strategy for migratory DC in MLN within live singlet cells. (C-F) Expression of CCR7, CD86, PDL2 and ICOSL on migratory DC in MLN measured as geometric mean fluorescence intensity. (G) Gating strategy for Treg within live singlet cells. (H-I) Percentage of Treg within live singlet lymphocytes in MLN and lung. (J-M) Expression of GITR, ICOS, IL-10 on lung Treg measured as geometric mean fluorescence intensity and % CTLA-4 ^+^ Treg. * P<0.05 and ** P<0.01 using Kruskal-Wallis with Dunn’s post-test. Each symbol represents one mouse and mean values are indicated. This pilot experiment was performed once. Insufficient cells were obtained from one mouse in the ND/OVA group to perform the staining for Fig 2C, E.

We next performed a pilot experiment to investigate the effect of HFD on induction of respiratory tolerance. Respiratory tolerance was induced by administering OVA intranasally (i.n.), or saline (SAL) as a control, daily for 5 days (Fig 2A). After 24 h, we collected lung and mediastinal lymph nodes (MLN) and examined the activation of DC, macrophages and Treg, cell populations associated with the establishment of respiratory tolerance [21–23]. This experiment was performed once, therefore the results are only indicative. After OVA administration, the expression of the LN-homing chemokine receptor CCR7 and the activation marker CD86 showed a tendency to be up-regulated on MLN migratory DC (CD11c^hi^ MHCII^hi^ [24]) in mice fed ND but not HFD (Fig 2B–2D). The expression of programmed cell death ligand 2 (PDL2) and inducible T-Cell Co-Stimulator Ligand (ICOSL) tended to be higher in ND compared to HFD mice, after OVA and saline administration respectively (Fig 2E and 2F). Since CD86, ICOSL and PDL2 have been shown previously to be essential for induction of tolerance [9,22,25], these results are suggestive of an altered tolerance in HFD mice.

Similar changes were present in the lung. With OVA administration there was a trend for increased percentage of DC (CD11c^+^ MHCII^hi^) in ND mice, and alveolar macrophages (auto-fluorescent CD11c^+^ F4/80^+^) in both diets, with greater macrophage proportions in the HFD group after PBS and OVA instillations (S1A, S1B, S1E and S1F Fig). The activation of macrophages, measured as expression of MHCII, CD80 and CD86 on the surface of the cells, showed a propensity to decrease after OVA compared to saline, and this appeared more pronounced in HFD compared to ND fed mice (S1G and S1I Fig). The percentage of CD40^+^ DC and the level of CD80 on DC tended to be higher in ND compared to HFD mice (S1C and S1D Fig), after OVA or saline. While the total number of lung cells was not affected by the instillation of OVA (data not shown), the percentage of macrophages and B cells was inclined to be affected by tolerance induction, with a trend toward increased macrophage and decreased B cell proportions (S1F and S1J Fig). Other cell types involved in asthma i.e. eosinophils and neutrophils were not affected by tolerance (S1J Fig). These preliminary findings suggest that a different activation of APCs might occur in MLN and lung after tolerance in ND versus HFD mice, while there was no difference at steady state. Further indication of this phenomenon was observed after induction of AAI.

To test whether diet might affect the T cell stimulatory capacity of APC, we co-cultured OVA-loaded APC from lung and MLN of naïve ND or HFD mice with CFSE-labeled OVA-specific OT-II CD4^+^CD25^-^ T cells. After 84 h, we determined the proportion of CFSE^low^ T cells in the culture, which underwent at least one proliferation cycle. MLN DC induced the strongest proliferation of OVA-specific CD4^+^ T cells, while lung macrophages induced proliferation weakly, but there was no difference between ND and HFD groups (S1K Fig). We observed a trend for reduced T cell stimulatory capacity by lung DC from HFD mice compared to ND mice, although this did not reach significance (S1K Fig).

We then examined the possibility that diet may affect APC allergen uptake and/or migration following OVA administration by instilling fluorescent OVA intratracheally (S1L Fig). We observed that i.n. OVA administration decreased subsequent fluorescent OVA uptake by lung and MLN DC, more pronouncedly so in the HFD group (S1M–S1Q Fig). However, fluorescent OVA uptake was not affected by diet. There were saturating levels of allergen-positive alveolar macrophages, regardless of diet or tolerance status (S1M Fig).

Given the important role for Treg in immune regulation and maintenance of respiratory tolerance [22,23], we investigated the effect of i.n. OVA on Treg proportions and activation in the same pilot study. In MLN, the frequency of Treg (CD4^+^FoxP3^+^CD25^+^) showed a tendency to decrease after OVA, and this change was more pronounced in ND than HFD mice (Fig 2G and 2H). In contrast, in the lung, OVA had a tendency to increase the percentage of Treg in both diets (Fig 2I). Moreover, OVA tended to increase the expression of glucocorticoid-induced tumor necrosis factor receptor (GITR, a molecule linked with Treg suppressive function [22]), ICOS, IL-10 and CTLA-4 on lung Treg in mice fed ND, but not HFD (Fig 2J–2M). Collectively, these preliminary findings suggest that HFD impairs the activation of DC and Treg induced by OVA instillation.

### HFD does not affect the ability of respiratory tolerance to inhibit allergic airway inflammation, but inhibits Th2 cytokine production

**Fig 3:**
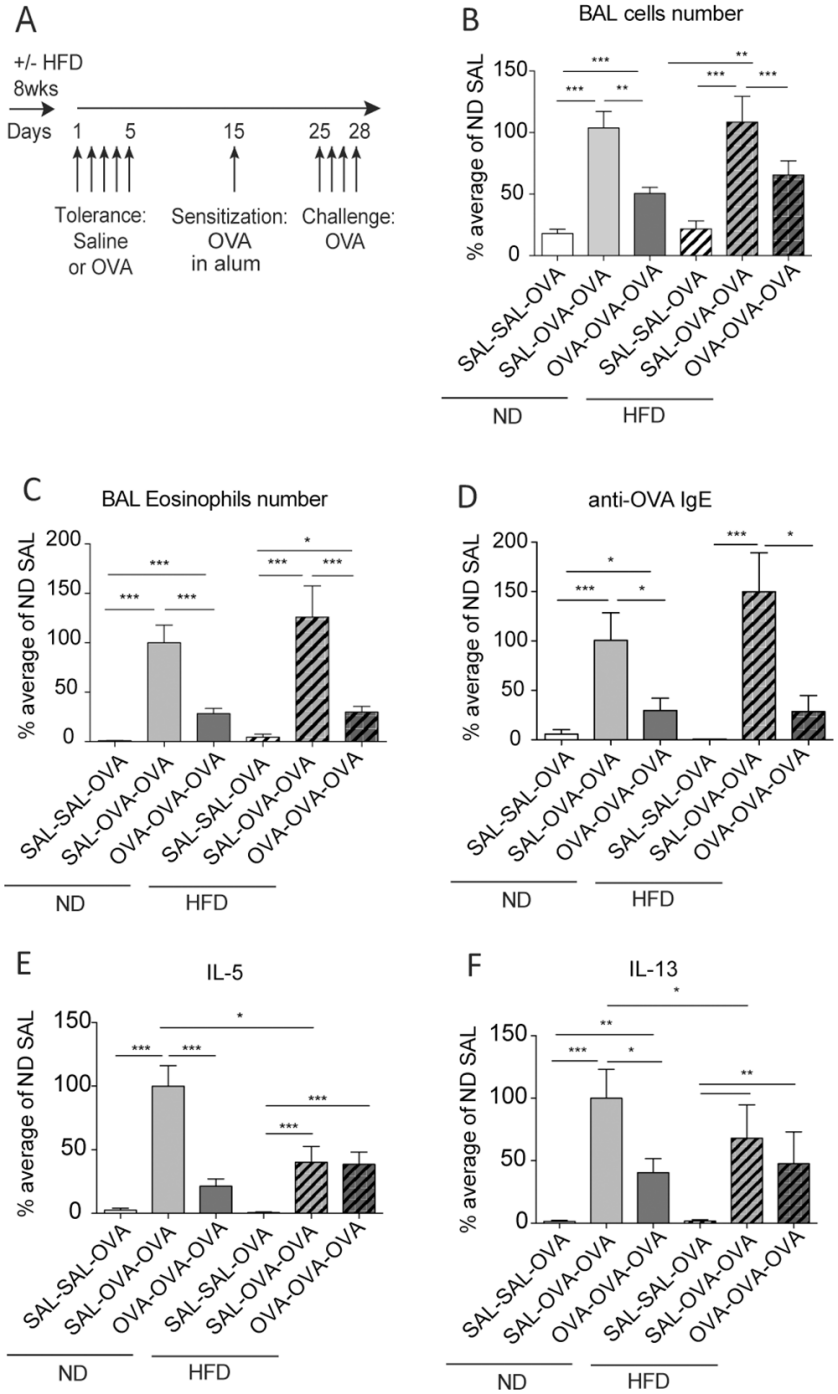
HFD impaired the T cell cytokine response but not the induction of respiratory tolerance. (A) Experimental design for diet regimen, respiratory tolerance and AAI. In this figure, all mice received OVA in alum i.p. and OVA challenge but differed with respect to the i.n. instillation prior to sensitization being OVA or saline, indicated as Tol. (B) Bronchoalveolar lavage (BAL) cell numbers, (C) BAL eosinophil number, (D) anti-OVA IgE in serum, (E-F) IL-5 and IL-13 measured in the supernatant of MLN cells restimulated with OVA *in vitro*. (B-F) The data are normalized to the average of the ND non-tolerized group (SAL/OVA/OVA), which was set at 100%. Mean ± SEM for mice from 3–5 independent experiments are shown. (B-C) N = 25–30 mice/group, (D) N = 7–9 mice/group, (E-F) N = 19–26 mice/group. * P<0.05, ** P<0.01 and *** P<0.001 using Kruskal-Wallis with Dunn’s post-test.

The reduction in DC and Treg activation following i.n. OVA instillation in mice fed with HFD suggests a possible defect in the development of respiratory inhalation tolerance and the capacity to dampen the allergic reaction to OVA. We tested this by inducing respiratory tolerance with i.n. OVA, or saline as control, and eliciting AAI by sensitizing mice i.p. with OVA in alum, or saline as control, and challenging i.n. with OVA (Fig 3A). To facilitate comparison of results from different experiments, original values were converted to a percentage relative to the reference group, the ND SAL tolerized allergic group (SAL/OVA/OVA). The original values from a representative experiment are portrayed in S2 Fig.

OVA instillation prior to sensitization reduced cell infiltration in BAL and lung in both ND and HFD mice (Fig 3B and S2A–S2C Fig). In particular, it reduced eosinophilia, typical of this model, in both BAL and lung (Fig 3C and S2A, S2D and S2E Fig). Similarly, serum OVA-specific IgE production was inhibited by OVA tolerance in mice fed both diets (Fig 3D and S2F Fig).

Of 25 molecules tested, nine cytokines and chemokines were upregulated in BAL fluid of ND mice following sensitization and challenge with OVA compared to mock sensitized mice (IL-4, IL-5, IL-13, IL-6, IFN-γ, IL-12p40, MCP-1, GM-CSF and MIP-1α) (S2G Fig). Levels of most of these cytokines were partially or totally reduced in mice that received i.n. OVA prior to sensitization (S2G Fig). However, no significant difference in the concentration of these cytokines was found between the diets, either in the SAL tolerized or the OVA tolerized group (S2G Fig). The other 16 cytokines tested were not affected by diet or allergic sensitization (data not shown) except for TSLP, which was significantly upregulated in HFD mice compared to ND mice (S2G Fig). However, TSLP expression was not affected by allergic sensitization relative to the saline control group (S2G Fig). Since it was shown recently that IL-17 is central in the development of non-allergic asthma in obese mice and responsible for abrogating respiratory tolerance [26,27], we measured IL-17A concentration in serum and BAL fluid of mice: IL-17A was increased in the serum after AAI in both diets and reduced in tolerized mice, mainly in HFD (S2H Fig). Neither diet nor allergic sensitization affected the concentration of IL-17A in BAL fluid (data not shown). No difference in AHR was detected between any of the groups (S2I Fig).

In the MLN, OVA instillation prior to sensitization inhibited the production of the Th2 cytokines IL-5 and IL-13, in the supernatant of MLN cells restimulated with OVA *in vitro* in the ND group (Fig 3E, 3F and S2J, S2K Fig). However, OVA tolerance did not decrease IL-5 and IL-13 production in HFD mice, most likely because the concentrations of the cytokines were already very low (Fig 3E, 3F and S2J, S2K Fig). Moreover, the level of these cytokines in the SAL tolerized HFD group was significantly lower than that in the ND group (Fig 3E, 3F and S2J, S2K Fig).

Taken together, these results show that HFD impaired the Th2 cytokine response to OVA following induction of AAI, but did not affect induction of the pulmonary allergic inflammatory response itself or the capacity to develop respiratory tolerance.

### HFD impairs DC activation after allergic airway inflammation

To understand how HFD affected the Th2 response to OVA, we investigated DC, macrophage and Treg populations in lung and MLN after respiratory tolerance and AAI. As outlined above, original values were converted to a percentage relative to a reference group, the ND SAL tolerized allergic group (SAL in the figure) to compare results from different experiments. The original values from a representative experiment are portrayed in S3 Fig.

**Fig 4:**
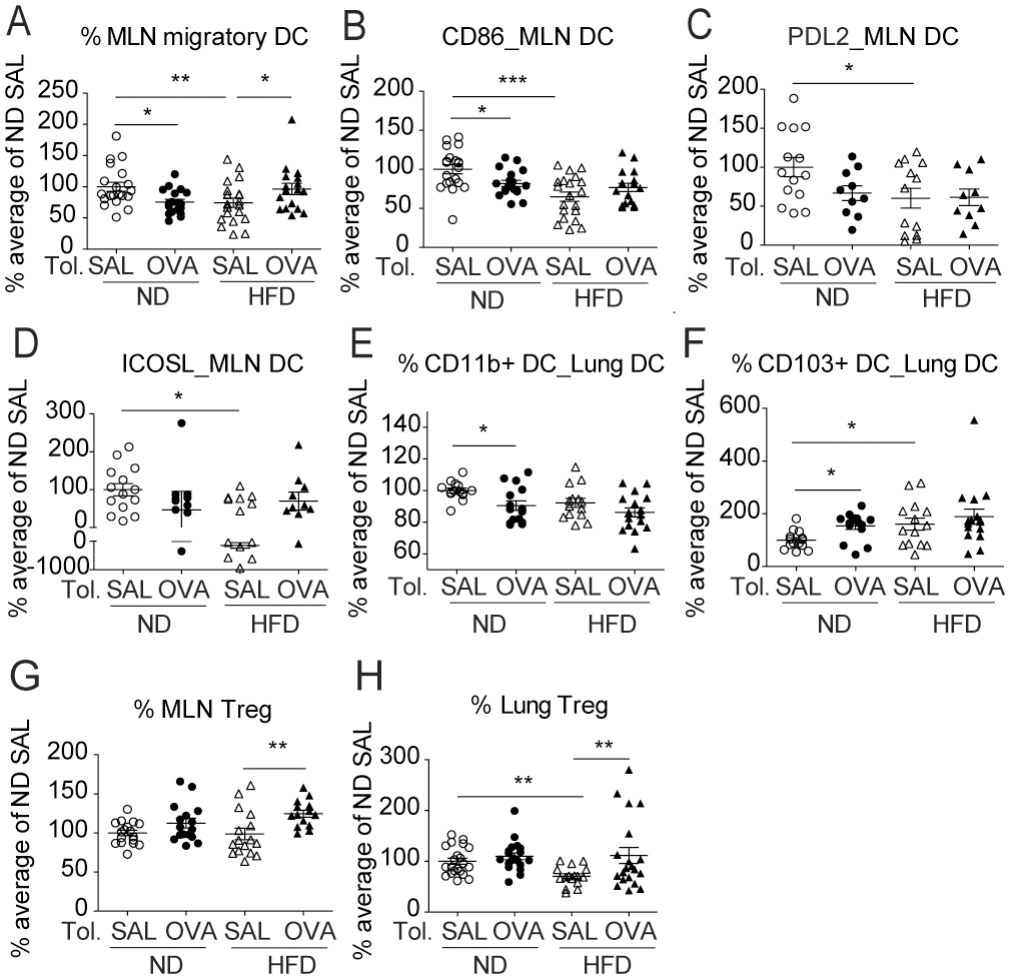
HFD decreased the percentage and activation of migratory DC and Treg after tolerance and AAI. Analysis of MLN and lung after respiratory tolerance and AAI as per Fig 3A. DC are gated as per Fig 2B. (A) Percentage of migratory DC in the MLN and (B-D) geometric mean fluorescence intensity of CD86, PDL2 and ICOSL on MLN migratory DC. (E-F) Percentage of CD11b^+^ and CD103^+^ among lung DC. Treg are gated as per Fig 2G. (G-H) Percentage of Treg in MLN and in lung. The data are normalized to the average of the ND non-tolerized (SAL) group, which was set at 100%, and data for 2–3 independent experiments are pooled. Each symbol represents one mouse and mean ± SEM are indicated. N = 17-20/group (A-B), N = 10-13/group (C-D), N = 14-16/group (E-F), n = 14-17/group (G-H). *P<0.05, **P<0.01, ***P<0.001 using one-way ANOVA with Bonferroni’s post-test.

In MLN, OVA instillation prior to sensitization decreased the percentage of migratory DC and their expression of the co-stimulatory molecule CD86 in ND mice, and a similar trend were detected for PDL2 and ICOSL (Fig 4A–4D, S3A–S3D Fig). These changes reflected the capacity of respiratory tolerance to prevent the activation of DC in MLN, decreasing the levels of these molecules to levels seen for SAL sensitized mice (S3A–S3D Fig). In contrast to ND mice, HFD mice showed slightly increased frequency of migratory DC and expression of CD86 and ICOSL in OVA tolerized compared to SAL tolerized mice (Fig 4A–4D). Notably, mice in the HFD SAL tolerized group had reduced percentage of migratory DC and expression of CD86, ICOSL and PDL2 compared to the ND SAL tolerized group (Fig 4A–4D), consistent with the reduced cytokine production observed in the MLN (Fig 3D and 3E).

In the lung of ND mice, OVA tolerance decreased slightly the percentage of DC (S3E and S3F Fig). Interestingly, OVA tolerance significantly decreased proportions of lung ‘inflammatory’ CD11b^+^ DC in the ND group (Fig 4E, S3G Fig) suggested in the literature to drive AAI [24]. OVA Tolerance also increased the proportions of putative ‘tolerogenic’ lung CD103^+^ DC that are unable to induce Th2 cells [24,28], in the ND group, while a similar but non-significant trend was seen in HFD mice (Fig 4F, S3H Fig). The SAL tolerized HFD group had an increased proportion of CD103^+^ DC compared to the SAL tolerized ND group, suggesting a higher anti-inflammatory capacity in the HFD lung and consistent with the lower cytokine response in the MLN (Fig 4G, S3H Fig). However, in contrast to the results for the MLN, there was no difference in the expression of the activation markers PDL2, CD86 and ICOSL on lung DC between the ND and HFD groups (S3I–S3L Fig). Of note, the frequency of lung macrophages, implicated in the establishment of respiratory tolerance [23], was increased in OVA tolerized groups and showed a decreased expression of PDL2 compared to SAL tolerized groups, indicating reduced activation status (S3M–S3P Fig). However, these changes were comparable in both diets (S3M–S3P Fig).

Tolerance increased the percentage of Treg in MLN and the lung, although this effect was more profound in the HFD than the ND group (Fig 4G–4I, S3Q and S3R Fig). The reduced percentage of lung Treg in the SAL tolerized HFD group compared to SAL tolerized ND group (Fig 4H) is consistent with a lower inflammatory response in the lung of HFD mice, as Treg increase in the lung after AAI (data not shown). In conclusion, HFD reduced DC migration and activation in the MLN after AAI, consistent with lower Th2 cytokine production in the MLN.

## Discussion

In this report we show that a HFD can inhibit the activation of DC and T cells after the induction of respiratory tolerance and AAI. Mice fed a HFD had a marked decrease in Th2 cytokine production following induction of AAI, corroborating previous reports [29], and supporting the hypothesis that obesity skews the immune response away from the allergic Th2 type response [30]. This skewing is thought to be due to a chronic inflammatory Th1/Th17 type milieu during obesity, with increased IL-6, TNF-α and IFN-γ [31,32]. In support of this view, it was recently shown that HFD increases the risk and severity of non-allergic asthma due to a heightened Th17/IL-17 response [26], which might explain why obesity (commonly as a result of increased fat consumption in the diet) in humans consistently increases the risk of developing non-allergic asthma [3,4], while the correlation with allergic asthma is tenuous [5,6]. This low-grade Th1/Th17-biased chronic inflammation might increase the threshold of ‘reprogramming’ DC to a Th2-biased state [33]. While we could not detect a difference in IL17 and IL6 expression between HFD and ND mice, we observed a reduction in DC activation and a concomitant decrease in Th2 cytokines in the MLN of HFD mice compared with the ND group after AAI. It is likely that the decreased DC activation accounts for the reduction in T cell cytokine production. Moreover, the proportion of putative tolerogenic CD103^+^ DC [28] was increased in HFD mice compared with ND mice after AAI, suggesting a higher anti-inflammatory capacity in HFD mice, consistent with their lower cytokine response. However, despite the lower Th2 cytokine production and inhibition of DC activation, the lung inflammation during AAI (total leukocytes and eosinophils in both BAL and lung) was not reduced in HFD mice. Our results corroborate previous findings that HFD does not affect the severity of experimental allergic asthma, and are consistent with human studies showing that obesity does not increase the risk of developing allergic asthma [5,29].

A key aim of our work was to investigate whether, and if so how, HFD affects respiratory tolerance. Our pilot study data suggest that immediately after intranasal OVA administration during tolerization, DC and Treg tend to increase their expression of activation markers and that this upregulation was not present in HFD mice. This pilot study also supports the concept that alveolar macrophages are involved in respiratory tolerance induction [23] as their numbers and activation tended to be modulated by tolerance, although these cells were not affected by diet. Consistent with tolerance increasing the activation of lung and MLN APC, we observed a tendency for increased recruitment and activation of lung Treg after tolerance induction in ND mice, and noted that Treg activation (but not recruitment) tended to be decreased in HFD compared to ND mice. Nevertheless, despite the indication of a lower activation of DC and Treg after tolerance, HFD mice developed an efficient respiratory tolerance, reflected by reduced airway inflammatory cell infiltration and anti-OVA Ab production after AAI. It is possible that the lower expression levels of co-stimulatory markers on DC and activation molecules on Treg in HFD mice were still sufficient to initiate successful tolerization. It is also possible that tolerance could not reduce the already low Th2 cytokine production in HFD mice after AAI.

An essential APC function is the uptake of foreign antigens and we therefore sought to investigate whether this was affected by tolerance. Tolerance induction reduced subsequent OVA uptake by DC in the MLN and lung, and this was notably more pronounced in the HFD group. This reduction was unlikely to be due to saturation, as nearly 100% of alveolar macrophages took up OVA in the lung after tolerance, but is consistent with DC maturation, with concomitant reduced capacity to process and present proteins [34]. DC maturation following intranasal OVA instillation, as shown by expression of CD86, ICOSL and PDL2, could occur as a consequence of exposure to LPS [34], although the OVA we used contained low endotoxin levels (40EU/100μg). Nevertheless, the fact that pre-exposure to OVA containing low levels of endotoxin did not inhibit the induction of respiratory tolerance suggests that the DC maturation we observed was insufficient to prime an anti-OVA adaptive immune response. However, HFD alone did not affect the uptake of OVA in the lung, suggesting that the intrinsic phagocytic capacity of the cells was not affected by diet.

Obesity is a complex pathophysiological state where alterations to several metabolic and immune pathways interplay. The influence of obesity and diet on immune responses has been shown to be mediated by hormonal and cytokine imbalances [26,35], alteration of gut microbiota [36], and their metabolites [37]. Changes to the gut microbiota composition due to changes in diet disturb gut immune homeostasis [38], and can contribute to low-grade inflammation [39] and metabolic disorders [18,36]. In our study, the changes in the gut microbiota of HFD mice were small compared to previous findings, despite a similar percentage of fat being used in the diet [36]. Nevertheless, we found an increase in *Firmicutes* bacteria and a decrease in *Bacteroides* in HFD mice, as reported previously for studies that used a similar percentage fat in the diet [36,40]. Consistent with other reports [18], we also found a decreased proportion of the genus *Akkermansia* in HFD mice, species that contribute to control of HFD-induced metabolic disorders [18]. We postulate that the small differences in microbiota after HFD could be due to the duration of the diet. Extensive research has focused on how the gut microbiota can influence and, with modification, alleviate obesity symptoms [41]. Changes in the gut microbiota induced by HFD have been shown to result in a higher concentration of LPS in the blood due to increased gut permeability, which is thought to contribute to low-grade inflammation [39]. It has been shown that LPS exposure or triggering of TLR4 reduces AAI and allergic asthma by suppressing Th2 cell stimulation by DC [42,43]. Therefore, the reduction in Th2 cytokines in HFD mice could have resulted from the high concentration of LPS in the blood due to a leaky gut. However, this hypothesis was not confirmed in our experiments, where the level of LPS in the serum of mice did not differ with type of diet or induction of tolerance or allergic airway inflammation (data not shown).

Another mechanism by which the gut microbiota influences the immune system in distal organs is through the production of metabolites, including SCFA [44]. SCFA have been shown to increase Treg frequency [45] and alleviate AAI [46], as well as improve insulin metabolism and decrease fat accumulation in obesity [47]. In our study, SCFA were decreased in fecal samples of HFD mice compared to ND group, suggesting that the lower Treg activation status in HFD mice could be due to a reduction in SCFA. However, these decreased SCFA levels were not associated with increased severity of airway inflammation in HFD mice, suggesting that the levels of SCFA in these mice were still sufficient to moderate the severity of symptoms.

There is emerging evidence that immune cell function is linked to, and regulated by, the ability to activate certain metabolic pathways (as reviewed in [20]). HFD has been shown to alter metabolism of adipocytes, kidney and muscle cells [19,48,49]. Therefore, we hypothesized that HFD diet could change the metabolic profile of lung cells. Indeed, we show that in the presence of glucose, the metabolic capacity of lung cells from HFD mice was higher compared to ND mice. These results are in agreement with those obtained for kidney cells and adipocytes [19,48], but opposite to those for skeletal muscle cells [49], suggesting that increased mitochondrial respiration of glucose can be a ubiquitous effect of adaptation to increased nutrient supply in non-muscle cells. In future studies it will be important to dissect which cell types in the lungs are affected by diet and to understand how these metabolic changes impact on their function.

In conclusion, we show that HFD feeding in mice induces significant weight gain, alters gut microbiota and gut metabolites. It also increases the glucose metabolic capacity of lung cells. Our findings in the murine system were consistent with human data investigating obesity and allergic responses [5,6] in showing that HFD allows development of respiratory tolerance and does not increase severity of allergic asthma. However, HFD inhibited the production of Th2 cytokines by T cells and possibly impaired allergen-induced activation of DC and Treg, potentially due to the interplay of gut microbiota and the immune system. Our findings provide further insight into the complex relationship between diet, microbiota and immunological responses which have implication in the management of obesity and its co-morbidities.

## Supporting Information

S1 Fig Analysis of expression of surface molecules on lung DC and macrophages after OVA tolerance and OVA uptake. (A) Gating strategy for lung DC within live singlet cells as CD11c^+^ autofluorescent^-^ MHCII^hi^ cells. (B) Percentage of lung DC within live singlet cells, (C) Percentage CD40^+^ cells within lung DC, (D) geometric mean fluorescence intensity of CD80 on lung DC. (E) Gating strategy for autofluorescent^+^ CD11c^+^ F4/80^+^ FSC-A^hi^ alveolar macrophages in live singlet lung cells and (F) percentage of alveolar macrophages, (G-I) geometric mean fluorescence intensity of CD80, CD86 and MHCII on lung macrophages. The pilot experiment was performed once. Mean ± SEM are indicated by the bars, *P<0.05, statistics calculated using Kruskal-Wallis with Dunn’s post-test. (J) Lung cellular composition as determined by flow cytometric analysis. *** P< 0.001 difference in macrophage and B cell percentage between SAL and OVA treated mice, calculated with 2-way ANOVA. Mean±SEM are indicated by the bars. (K) Percentage proliferating CD4^+^Va2^+^ OT-II T cells after 84 h of co-culture with OVA-pulsed MLN DC, lung DC or alveolar macrophages. Proliferating cells were gated for having half or less CFSE fluorescence than cells cultured with DC non-pulsed with OVA. The pooled data from two independent experiments are shown. (L) Experimental design of diet regimen, induction of respiratory tolerance via i.n. administration of saline or OVA, and i.t. administration of fluorescent AF594 OVA. (M) Percentage OVA-AF594^+^ alveolar macrophages, gated as per panel E, (N) percentage OVA-AF594^+^ lung DC, gated as panel A, and (O) geometric mean fluorescence intensity of OVA-AF594 in OVA^+^ lung DC. (P) Percentage OVA-AF594^+^ MLN migratory DC, gated as in Fig 2B, and (Q) geometric mean fluorescence intensity of OVA-AF594 in OVA^+^ MLN migratory DC. *P<0.05, **P<0.001 using Kruskal-Wallis with Dunn’s post-test. The experiment was performed once.
(TIF)

S2 Fig Diet does not affect induction of respiratory tolerance but impairs Th2 cytokine production. Tolerance and AAI were induced as in Fig 3A. SAL-SAL-OVA mice received saline during tolerance, were sensitized with saline in alum i.p. and challenged with ovalbumin. SAL-OVA-OVA mice received saline during tolerance, were sensitized with OVA in alum and challenged with OVA. OVA-OVA-OVA mice received OVA during the tolerance phase and then were sensitized with OVA in alum and challenged with OVA. (A) BAL differential counts, n = 4/group, representative of 3 experiments, statistics calculated on number of eosinophils. (B) Lung cell numbers as percentage of SAL/OVA/OVA and (C) lung cell numbers from 2 pooled independent experiments (n = 8/group). (D) Eosinophil cell numbers in the lung as percentage of SAL/OVA/OVA. (E) Percentage of APC and innate immune cells in lung of mice, data are pooled from 2 independent experiments, n = 8/group. Statistics calculated on percentage of eosinophils. (F) Anti-OVA IgE in serum as measured by ELISA. (G) Concentration of cytokines in BAL fluid measured with Biorad 23-plex and concentration of TSLP in BAL fluid by ELISA. (H) Concentration of IL-17A in serum. Mean values are indicated by the bars. (I) Airway resistance at different doses of methacholine, n = 8/group. (J-K) IL13 and IL5 measured in supernatant of MLN cells restimulated *in vitro* with OVA, only in groups sensitized to OVA. F, I-K: one representative experiment of 3 is shown. G-H: experiment performed once. Data in panels A, C, E, F-J are analyzed using Kruskall-Wallis and Dunn’s post-test. (B, D) The data are normalized to the average of the ND non-tolerized (SAL/OVA/OVA) group, which was set at 100% and 3 independent experiments are pooled, N = 19-21/group. Each symbol represents one mouse and mean and SEM are indicated. Data analyzed using one-way ANOVA and Bonferroni’s post-test. * P<0.05, **P<0.01, ***P<0.001.
(TIF)

S3 Fig Tolerance decreased DC and macrophage activation induced by AAI in MLN and lung. Tolerance and AAI were induced as in Fig 3A. SAL-SAL-SAL mice received saline during tolerance, saline in alum i.p. during AAI induction and were challenged with saline. SAL-OVA-OVA mice received saline during tolerance and were sensitized with OVA in alum and challenged with OVA. OVA-OVA-OVA mice received OVA during the tolerance phase and then were sensitized with OVA in alum and challenged with OVA. (A) % DC in MLN, gated as per Fig 2B. (B-D) Geometric mean fluorescence intensity of CD86, PDL2 and ICOSL on MLN DC. (E-F) Percentage lung DC gated as per S1A Fig. (G-H) Percentage of CD11b^+^ and CD103^+^ DC among all lung DC. (I-L) Geometric mean fluorescence intensity of PDL2, CD86 and ICOSL on lung DC. (M-P) Percentage alveolar macrophages, gated as in S1E Fig. (N-O) Geometric mean fluorescence intensity of PDL2 on lung macrophages. (Q and R) Percentage Treg in MLN and lung, gated as in Fig 2G. (A-D, F-H, J, N, P-R) One representative experiment out of 3 is shown; statistics calculated with Kruskall-Wallis with Dunn’s post-test. (E, I, K-M, O) The data are normalized to the average of the ND non-tolerized (SAL/OVA/OVA) group, which was set at 100% and 3 independent experiments are pooled, N = 19-21/group, N = 7–8 in J. Each symbol represents one mouse, and mean and SEM are indicated. * P<0.05, **P<0.01, ***P<0.001 using one-way ANOVA and Bonferroni’s post-test.
(TIF)

S1 Table Nutritional parameters of the diet. (DOCX)
