# Supplementary figures and images for: High Fat Diet Inhibits Dendritic Cell and T Cell Response to Allergens but Does Not Impair Inhalational Respiratory Tolerance
Source: PLoS One. 2016 Aug 2;11(8):e0160407. doi: 10.1371/journal.pone.0160407 (PMC4970708; doi:10.1371/journal.pone.0160407)

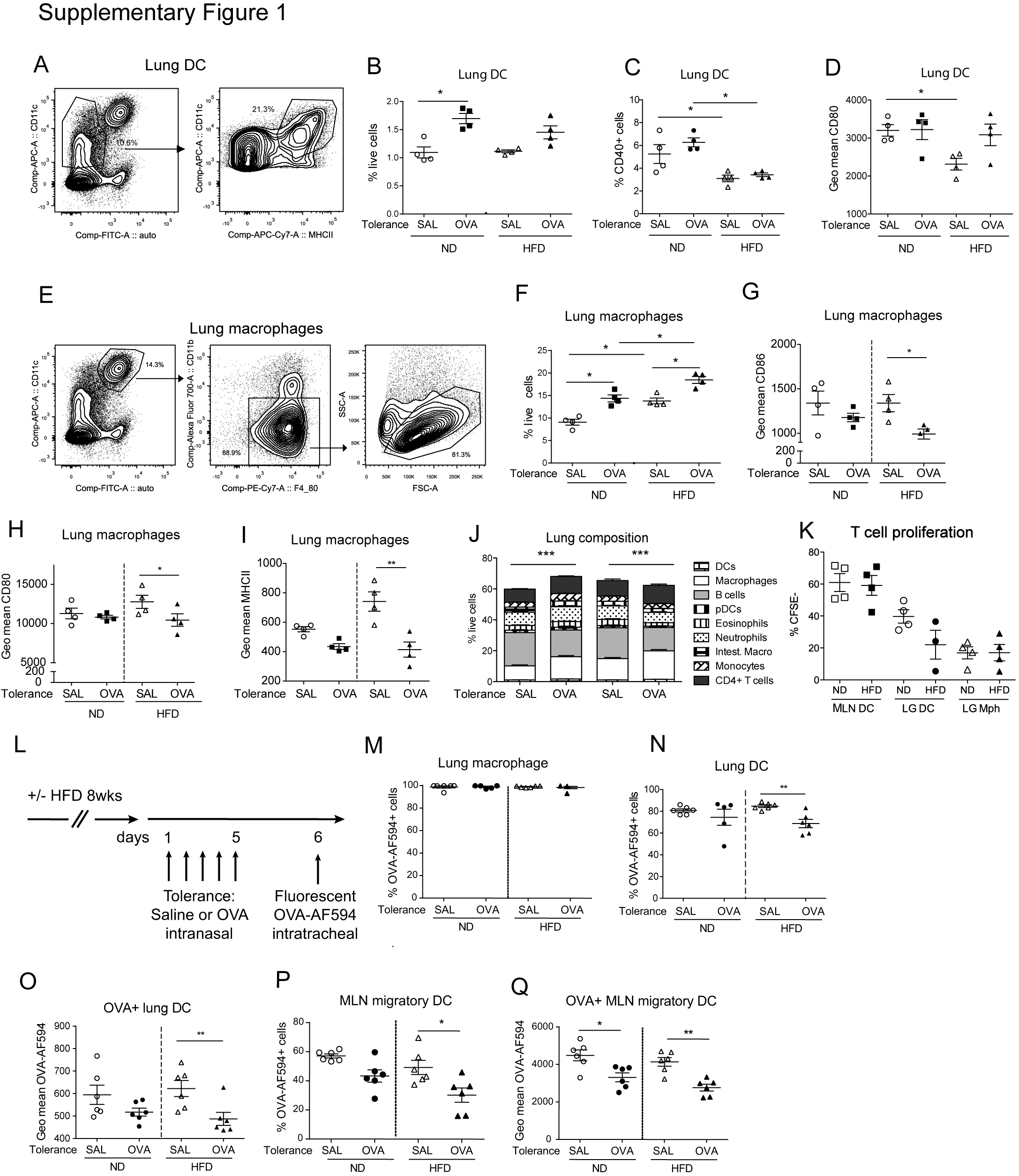

Supplement: S1 Fig — (A) Gating strategy for lung DC within live singlet cells as CD11c+ autofluorescent- MHCIIhi cells. (B) Percentage of lung DC within live singlet cells, (C) Percentage CD40+ cells within lung DC, (D) geometric mean fluorescence intensity of CD80 on lung DC. (E) Gating strategy for autofluorescent+ CD11c+ F4/80+ FSC-Ahi alveolar macrophages in live singlet lung cells and (F) percentage of alveolar macrophages, (G-I) geometric mean fluorescence intensity of CD80, CD86 and MHCII on lung macrophages. The pilot experiment was performed once. Mean ± SEM are indicated by the bars, *P<0.05, statistics calculated using Kruskal-Wallis with Dunn’s post-test. (J) Lung cellular composition as determined by flow cytometric analysis. *** P< 0.001 difference in macrophage and B cell percentage between SAL and OVA treated mice, calculated with 2-way ANOVA. Mean±SEM are indicated by the bars. (K) Percentage proliferating CD4+Va2+ OT-II T cells after 84 h of co-culture with OVA-pulsed MLN DC, lung DC or alveolar macrophages. Proliferating cells were gated for having half or less CFSE fluorescence than cells cultured with DC non-pulsed with OVA. The pooled data from two independent experiments are shown. (L) Experimental design of diet regimen, induction of respiratory tolerance via i.n. administration of saline or OVA, and i.t. administration of fluorescent AF594 OVA. (M) Percentage OVA-AF594+ alveolar macrophages, gated as per panel E, (N) percentage OVA-AF594+ lung DC, gated as panel A, and (O) geometric mean fluorescence intensity of OVA-AF594 in OVA+ lung DC. (P) Percentage OVA-AF594+ MLN migratory DC, gated as in Fig 2B, and (Q) geometric mean fluorescence intensity of OVA-AF594 in OVA+ MLN migratory DC. *P<0.05, **P<0.001 using Kruskal-Wallis with Dunn’s post-test. The experiment was performed once. (TIF) [file pone.0160407.s001.tif]

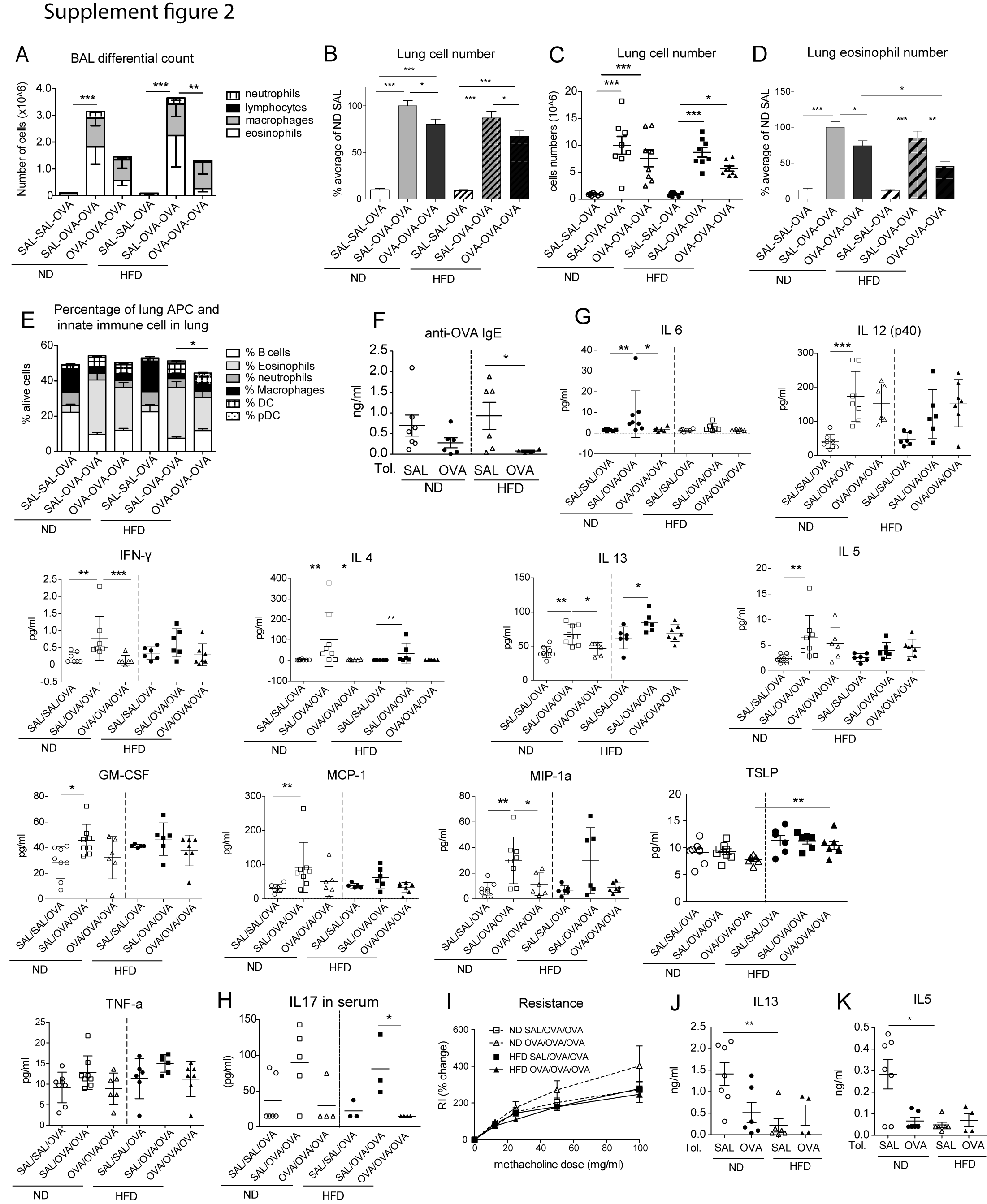

Supplement: S2 Fig — Tolerance and AAI were induced as in Fig 3A. SAL-SAL-OVA mice received saline during tolerance, were sensitized with saline in alum i.p. and challenged with ovalbumin. SAL-OVA-OVA mice received saline during tolerance, were sensitized with OVA in alum and challenged with OVA. OVA-OVA-OVA mice received OVA during the tolerance phase and then were sensitized with OVA in alum and challenged with OVA. (A) BAL differential counts, n = 4/group, representative of 3 experiments, statistics calculated on number of eosinophils. (B) Lung cell numbers as percentage of SAL/OVA/OVA and (C) lung cell numbers from 2 pooled independent experiments (n = 8/group). (D) Eosinophil cell numbers in the lung as percentage of SAL/OVA/OVA. (E) Percentage of APC and innate immune cells in lung of mice, data are pooled from 2 independent experiments, n = 8/group. Statistics calculated on percentage of eosinophils. (F) Anti-OVA IgE in serum as measured by ELISA. (G) Concentration of cytokines in BAL fluid measured with Biorad 23-plex and concentration of TSLP in BAL fluid by ELISA. (H) Concentration of IL-17A in serum. Mean values are indicated by the bars. (I) Airway resistance at different doses of methacholine, n = 8/group. (J-K) IL13 and IL5 measured in supernatant of MLN cells restimulated in vitro with OVA, only in groups sensitized to OVA. F, I-K: one representative experiment of 3 is shown. G-H: experiment performed once. Data in panels A, C, E, F-J are analyzed using Kruskall-Wallis and Dunn’s post-test. (B, D) The data are normalized to the average of the ND non-tolerized (SAL/OVA/OVA) group, which was set at 100% and 3 independent experiments are pooled, N = 19-21/group. Each symbol represents one mouse and mean and SEM are indicated. Data analyzed using one-way ANOVA and Bonferroni’s post-test. * P<0.05, **P<0.01, ***P<0.001. (TIF) [file pone.0160407.s002.tif]

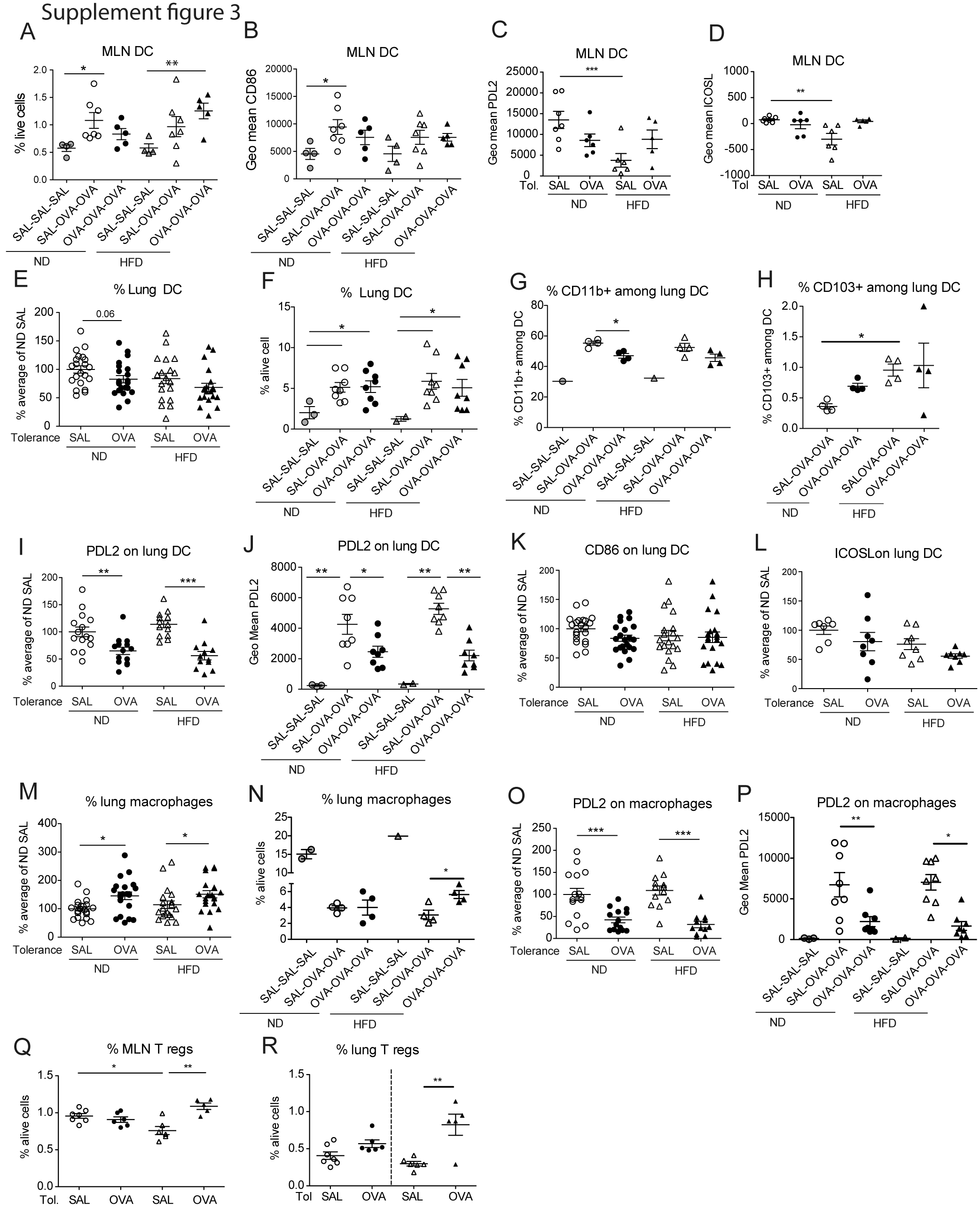

Supplement: S3 Fig — Tolerance and AAI were induced as in Fig 3A. SAL-SAL-SAL mice received saline during tolerance, saline in alum i.p. during AAI induction and were challenged with saline. SAL-OVA-OVA mice received saline during tolerance and were sensitized with OVA in alum and challenged with OVA. OVA-OVA-OVA mice received OVA during the tolerance phase and then were sensitized with OVA in alum and challenged with OVA. (A) % DC in MLN, gated as per Fig 2B. (B-D) Geometric mean fluorescence intensity of CD86, PDL2 and ICOSL on MLN DC. (E-F) Percentage lung DC gated as per S1A Fig. (G-H) Percentage of CD11b+ and CD103+ DC among all lung DC. (I-L) Geometric mean fluorescence intensity of PDL2, CD86 and ICOSL on lung DC. (M-P) Percentage alveolar macrophages, gated as in S1E Fig. (N-O) Geometric mean fluorescence intensity of PDL2 on lung macrophages. (Q and R) Percentage Treg in MLN and lung, gated as in Fig 2G. (A-D, F-H, J, N, P-R) One representative experiment out of 3 is shown; statistics calculated with Kruskall-Wallis with Dunn’s post-test. (E, I, K-M, O) The data are normalized to the average of the ND non-tolerized (SAL/OVA/OVA) group, which was set at 100% and 3 independent experiments are pooled, N = 19-21/group, N = 7–8 in J. Each symbol represents one mouse, and mean and SEM are indicated. * P<0.05, **P<0.01, ***P<0.001 using one-way ANOVA and Bonferroni’s post-test. (TIF) [file pone.0160407.s003.tif]
