# Supplementary material for: High Fat Diet Inhibits Dendritic Cell and T Cell Response to Allergens but Does Not Impair Inhalational Respiratory Tolerance
Source: PLoS One. 2016 Aug 2;11(8):e0160407. doi: 10.1371/journal.pone.0160407 (PMC4970708; doi:10.1371/journal.pone.0160407)
Supplement: S1 Table — (DOCX) [file pone.0160407.s004.docx]

Supplemental Table 1: Nutritional parameters of the diets

| **Diet** | Digestible energy | Fat | Fibre | Protein | Energy from protein | Energy from lipids | Energy from carbohydrates |
| --- | --- | --- | --- | --- | --- | --- | --- |
| **ND** | 13-14 MJ/Kg | 5-9% | 5-10% | 20-22% | 22% | 12-24% | 48-64% |
| **HFD** | 19 MJ/Kg | 23.5% | 5.9% | 22.6% | 21% | 43% | 36% |
